# CRISPR deactivation in mammalian cells using photocleavable guide RNAs

**DOI:** 10.1016/j.xpro.2021.100909

**Published:** 2021-10-20

**Authors:** Roger S. Zou, Yang Liu, Taekjip Ha

**Affiliations:** 1 Department of Biomedical Engineering, Johns Hopkins University School of Medicine, Baltimore, MD 21205, USA; 2 Department of Biophysics and Biophysical Chemistry, Johns Hopkins University School of Medicine, Baltimore, MD 21205, USA; 3 Department of Biophysics, Johns Hopkins University, Baltimore, MD 21205, USA; 4 Howard Hughes Medical Institute, Baltimore, MD 21205, USA

**Keywords:** Cell Biology, Cell-based Assays, Genomics, Sequencing, Molecular Biology, CRISPR, Molecular/Chemical Probes, Biotechnology and bioengineering

## Abstract

The ability to deactivate CRISPR-Cas systems on demand would improve the safety and applicability of genome editing. Here, we detail a protocol using photocleavable guide RNAs (pcRNAs) to deactivate CRISPR-Cas9 inside cells. We verify that deactivation is both rapid and complete by checking for insertion-deletion (indel) mutations using Sanger sequencing. This protocol will be useful for researchers interested in using pcRNAs to improve genome editing specificity, characterize the timescales of genome editing, and study cellular DNA damage responses.

For complete details on the use and execution of this protocol, please refer to Zou et al. (2021).

## Highlights

• Protocol using photocleavable guide RNAs to deactivate CRISPR-Cas9 inside cells
• Deactivation is both rapid and complete by checking for insertion-deletion mutations
• Useful for characterizing genome editing dynamics and DNA repair processes

## Graphical abstract

**Figure:**
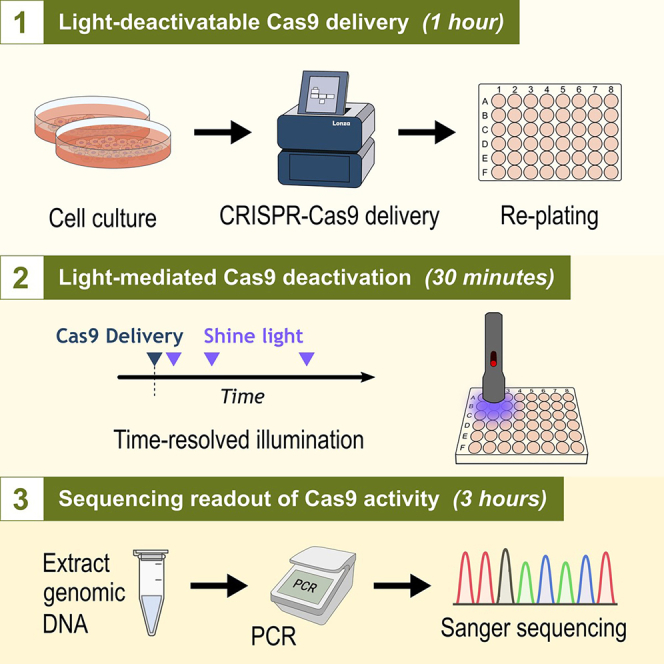

## Before you begin

The protocol below describes the specific steps using the Cas9 nuclease in HEK293T cells targeting a sequence near the *DNMT3B* gene. The gRNA sequence is called *HEK293 site 4* (or *HEK site 4*) (Hu et al., 2018). However, we have also used this protocol targeting other genomic sequences, with cytosine base editors and in K562 and induced pluripotent stem cells. Sanger sequencing is used for readout of Cas9 editing, but we have also used high-throughput amplicon sequencing with an Illumina MiSeq.

### Preparing oligonucleotides for polymerase chain reaction (PCR)

**Timing: 30 min**

1. Dilute dried oligonucleotide primers to make 100 μM stock in IDTE pH 8.
  a. Pulse spin tubes containing dried DNA so that the DNA is in the bottom of the tube.
  b. Add the expected volume of IDTE pH 8 into tubes containing dried primers (*HEKs4_F*, *HEKs4_R* from Table 1) to have a concentration of 100 μM.

**Table 1:** Synthetic DNA sequences

Name	Sequence (5′ to 3′)
TracrRNA	AGCAUAGCAAGUUAAAAUAAGGCUAGUCCGUUAUCAACUUGAAAAAGUG GCACCGAGUCGGUGCUUU
Pcl5_HEKsite4	GGCAC/iPC-Linker/GCGGCUGGAGGUGGGUUUUAGAGCUAUGCUGUUUUG
gRNA_HEKsite4	GGCACTGCGGCTGGAGGTGGGUUUUAGAGCUAUGCU
HEKs4_F	CCAGTGGTTCAATGGTCATCC
HEKs4_R	GGCCAGTGAAATCACCCTG
  c. Briefly vortex and pulse spin on a microfuge.
  d. Label tube accordingly.
2. Mix forward and reverse primers, then dilute to 5 μM with nuclease free water.
  a. Add 10 μL of 100 μM forward primer stock (*HEKs4_F*) to Eppendorf tube.
  b. Add 10 μL of 100 μM reverse primer stock (*HEKs4_R*) to Eppendorf tube.
  c. Add 180 μL of nuclease free water for 5 μM working stock (i.e., 10 μM of total oligonucleotide DNA), store in 4°C.
  d. Store 100 μM primer stocks in −20°C.

### Preparing Cas9 photocleavable crRNA (pcRNA) and tracrRNA

**Timing: 30 min**

3. Dilute dried RNA to make 100 μM stock in Nuclease Free Duplex Buffer.
  a. Pulse spin tubes containing dried RNA so that the RNA is in the bottom of the tube.
  b. Add the expected volume of Nuclease Free Duplex Buffer into two tubes containing dried photocleavable crRNA (the *Pcl5_HEKsite4* pcRNA from Table 1, targeting *DNMT3B*) and tracrRNA (Table 1) to each have a concentration of 100 μM.
  c. Briefly vortex and pulse spin on a microfuge.
  d. Label tube accordingly, store in −80°C.

**CRITICAL:** For light-sensitive pcRNAs, be sure to cover with foil as much as possible. Store covered in foil or in a black microcentrifuge tube.

### Preparing cells for electroporation

**Timing: 1–2 days**

4. Culture HEK293T cells following culture conditions in ATCC.
5. Ensure culture of 1–10 million cells at approximately 80% confluency at the time of electroporation.
6. Cells should also be routinely tested for mycoplasma infection.

## Key resources table

**Table:** 

REAGENT or RESOURCE	SOURCE	IDENTIFIER
**Chemicals, peptides, and recombinant proteins**		

Alt-R® S.p. Cas9 Nuclease V3	Integrated DNA Technologies	Cat#1081059
IDTE pH 8.0 (1**×** TE Solution)	Integrated DNA Technologies	Cat#11-05-01-13
Nuclease Free Duplex Buffer	Integrated DNA Technologies	Cat#11-01-03-01
Corning™ DMEM with L-Glutamine, Glucose, Sodium Pyruvate	Fisher Scientific	Cat#MT10013CV
Corning™ Regular Fetal Bovine Serum	Fisher Scientific	Cat#MT35011CV
Penicillin-Streptomycin	Thermo Fisher Scientific	Cat#15070063
3 M Sodium Acetate	Thermo Fisher Scientific	Cat# R1181
Trypsin-EDTA (0.05%), phenol red	Thermo Fisher Scientific	Cat#25300062
Collagen I, rat tail	Thermo Fisher Scientific	Cat#A1048301

**Critical commercial assays**		

SF Cell Line 4D-Nucleofector™ X Kit S (32 RCT)	Lonza	Cat#V4XC-2032
DNeasy Blood & Tissue Kit (50)	QIAGEN	Cat#69504
QIAquick PCR Purification Kit (50)	QIAGEN	Cat#28104
Q5® Hot Start High-Fidelity 2**×** Master Mix	New England BioLabs	Cat#M0494
E-Gel™ EX Agarose Gels, 2%	Invitrogen	Cat#G402022

**Experimental models: Cell lines**		

Human: HEK293T	ATCC	Cat#CRL-3216

**Oligonucleotides**		

Alt-R® CRISPR-Cas9 crRNA (sequence ‘gRNA_HEKsite4’ in Table 1)	Integrated DNA Technologies	N/A
Alt-R® CRISPR-Cas9 tracrRNA, 100 nmol	Integrated DNA Technologies	Cat#1072534
Alt-R® Cas9 Electroporation Enhancer, 10 nmol	Integrated DNA Technologies	Cat#1075916
tracrRNA (sequences in Table 1)	Integrated DNA Technologies	N/A
photocleavable crRNA (sequence ‘Pcl5_HEKsite4’ in Table 1)	Integrated DNA Technologies	N/A
HEKs4_F (sequences in Table 1)	Integrated DNA Technologies	N/A
HEKs4_R (sequences in Table 1)	Integrated DNA Technologies	N/A

**Software and algorithms**		

SnapGene Viewer	SnapGene	https://www.snapgene.com/snapgene-viewer/
TIDE	Brinkman et al. (2014)	http://shinyapps.datacurators.nl/tide/

**Deposited data**		

Original/source data in the paper is available	This paper	Mendeley Data https://data.mendeley.com/datasets/w55kctmzzc

**Other**		

4D-Nucleofector Core Unit	Lonza	Cat#AAF-1002B
4D-Nucleofector X Unit	Lonza	Cat#AAF-1002X
Nalgene™ Rapid-Flow™ Sterile Single Use Vacuum Filter Units	Thermo Fisher Scientific	Cat#09-741-02
Standard Hemocytometer	Weber Scientific	Cat#3048-12
C1000 Touch Thermal Cycler	Bio-Rad	Cat#1851148
Aluminum Foil	N/A	N/A
LED flashlight	JAXMAN	Cat#B06XW7S1CS (https://www.amazon.com/JAXMAN-Ultraviolet-365nm-Detector-Flashlight/dp/B06XW7S1CS/)
NanoDrop™ 2000 UV-Vis spectrophotometer	Thermo Fisher Scientific	Cat#ND-2000
E-Gel® iBase™ Power System with E-Gel™ Safe Imager™ Real-Time Transilluminator	Invitrogen	Cat#G6465

## Materials and equipment

**Lonza nucleofector system for electroporation of Cas9:** For delivery of Cas9/gRNA particles into HEK293T cells using electroporation, we use the Lonza nucleofector system. The Lonza system allows electroporation in different volumes (e.g., 20 μL or 100 μL), and previous literature have optimized the electroporation parameters for each cell line. This protocol is written for electroporation of 800,000 HEK293T cells in a 20 μL volume, using *‘SF Cell Line 4D-Nucleofector™ X Kit S’*. Alternative strategies include Neon transfection system (Thermo Fisher) or Gene Pulser Xcell (Bio-Rad).

**LED flashlight for deactivation:** We used a LED flashlight (JAXMAN) purchased from Amazon.com. There are many alternatives available on Amazon.com and other retailers. Ensure that the marketed wavelength is 365 nm.

**Bio-Rad Thermo Cycler for PCR:** To generate samples for Sanger sequencing, we performed PCR on the Bio-Rad C1000 Touch Thermo Cycler. Numerous commercial alternatives exist.

**Gel electrophoresis to evaluate PCR outcome:** We use E-Gel® EX Gel, 2% pre-cast gels on the E-Gel® iBase™ Power System/E-Gel™ Safe Imager™ Real-Time Transilluminator. Any gel electrophoresis system, including self-cast gels, will suffice.

**Table:** DMEM complete

Reagent	Final concentration	Amount
DMEM	89%	445 mL
Fetal Bovine Serum (FBS)	10%	50 mL
Penicillin-Streptomycin	1%	5 mL
**Total**	**100%**	**500 mL**

Store in 4°C, maximum time of 1 year.

***Optional:*** Use vacuum filter unit (Nalgene 0.2 μm Polyethersulfone) to ensure sterility of media before storage.

## Step-by-step method details

### Formation of Cas9/pcRNA ribonucleoprotein (RNP) complex

**Timing: 15 min**

This step forms the ribonucleoprotein (RNP) complex composed of Cas9 in complex with the light-deactivatable pcRNA and tracrRNA. This RNP is allowed to stably form before direct delivery into the cell.

1. Anneal light-deactivatable pcRNA with tracrRNA.
  a. Combine 1.2 μL pcRNA targeting *DNMT3B* with 1.2 μL tracrRNA (both at 100 μM in Nuclease Free Duplex Buffer) in PCR tube to form 2.4 μL of 50 μM annealed gRNA. Mix by pipetting up and down 5–10 times.
  b. Heat at 95°C in thermocycler for 3 min, then cool on benchtop (room temperature; 20°C) for 5 min.

**CRITICAL:** Cover tubes with aluminum foil whenever possible and be sure to avoid direct sun exposure. This would minimize unintended deactivation of pcRNA.

2. Formation of RNP complex and Lonza nucleofection mixture.
  a. Add Cas9 to annealed gRNA, mix by pipetting up and down 5–10 times, add PBS, then mix by pipetting again.

**Table:** 

Component	Volume
Annealed gRNA (50 μM)	2.4 μL
Cas9 (10 mg/mL)	1.7 μL
PBS	1.9 μL
**Total**	**5 μL**
  b. Cover the formed RNP complex with aluminum foil and leave at benchtop in room temperature (“RNP” in Figure 1A).

**Figure 1:**
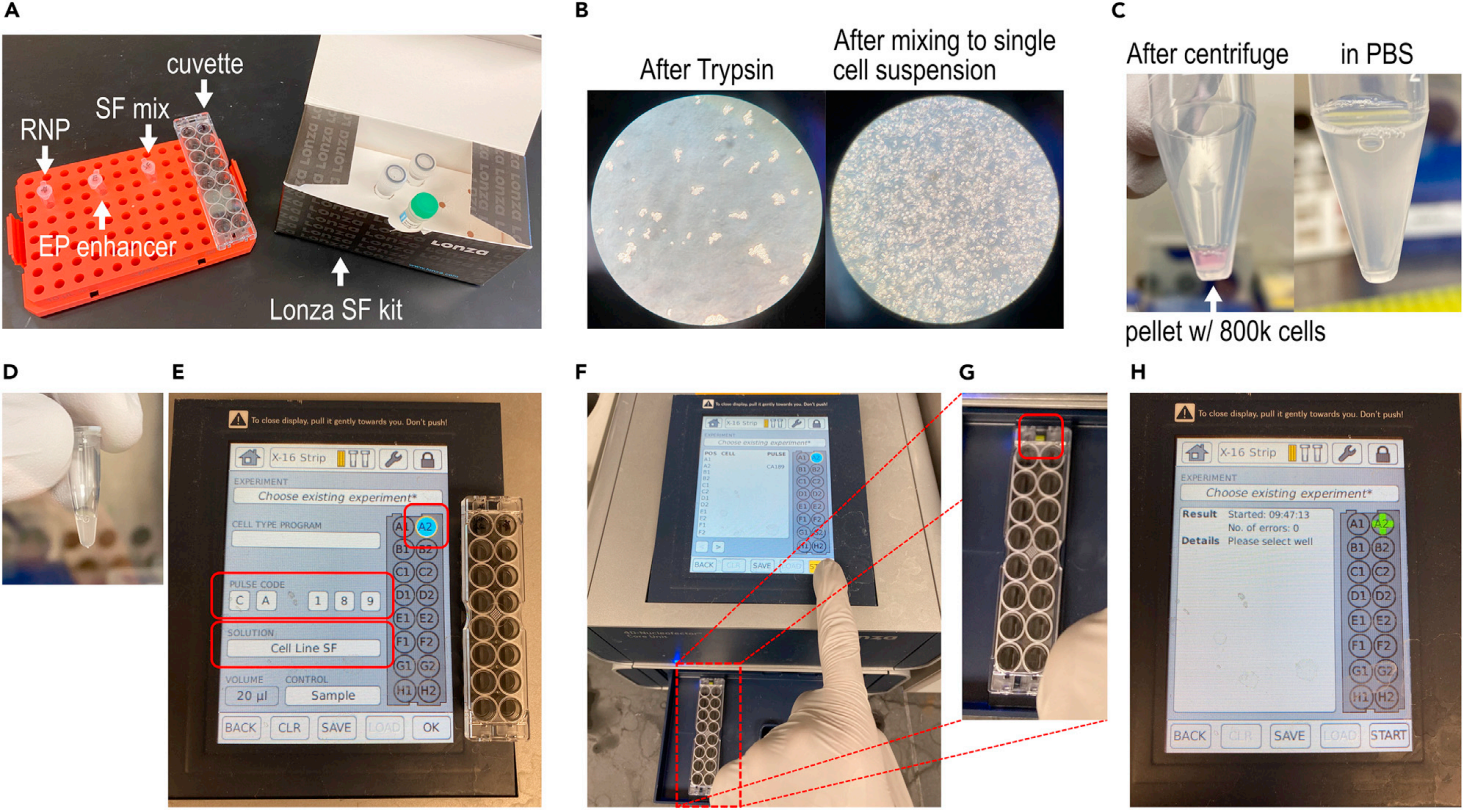
Cas9 electroporation protocol into HEK293T cells (A) Individual reagents necessary for electroporation, including Cas9/pcRNA RNP, EP enhancer, SF mix, and cuvette. (B) Dissociating the cells using Trypsin, and the cells after mixing to the required single cell suspension before electroporation. (C) After pelleting single cell suspension and resuspending in 1 mL PBS. (D) After mix cell pellet with RNP solution. (E) Electroporation settings on the Lonza electroporator. (F) Beginning the electroporation after placing the cuvette in the machine. (G) Zoom-in of the cuvette placement in the holder from panel f. Note the visible yellow pin that is visible only with the correct orientation (red box). (H) Screen display a successful electroporation.
  c. Open the Lonza SF kit (Figure 1A), which includes a cuvette, SF nucleofection reagent, and Supplement 1. In a separate tube, combine 16.4 μL of SF nucleofection reagent with 3.6 μL of Supplement 1 to form 20 μL of mixed nucleofection reagent (“SF mix” in Figure 1A). Mix by pipetting up and down 5–10 times.
  d. Proceed to prepare cells for electroporation in cell culture hood.

### RNP delivery into HEK293T cells

**Timing: 1 h**

This step delivers the Cas9/pcRNA RNP into HEK293T cells using electroporation (Liu et al., 2020). The RNP is first mixed thoroughly with the cells, then an electric pulse of optimal voltage is applied. Nanometer-scale holes in the cell membrane are formed, allowing the charged RNP particles to enter the cell. All steps are performed inside a tissue culture hood unless stated otherwise.

3. Prepare 48-well plates with collagen.
  a. Prepare 1 mL of 1:100 collagen solution by mixing 10 μL of collagen with 1 mL of ice-cold PBS.
  b. Add enough of 1:100 collagen to cover three wells of a 48-well plate (approximately 0.3 mL per well). Make sure the three wells are as spaced out as possible if on one plate. Incubate in 37°C cell culture incubator for 10 min.
    ***Note:*** To better ensure that deactivation light does not affect more than the intended well, we suggest using one well each in different 48-well plates. Here, we use multiple wells in one 48-well plate for ease of presentation. Other options include using black plates that prevent deactivation light penetration or reflection.
  c. Take out the plate from the incubator, move to inside the cell culture hood, remove the collagen solution, then let it dry in the cell culture hood with lid slightly ajar.

**CRITICAL:** Collagen-coated plates allow for rapid cell adherence after electroporation.

4. Prepare 800,000 HEK293T cells for electroporation.
  a. Heat DMEM complete, PBS, and 0.05% Trypsin-EDTA in 37°C water bath until the solutions reach 37°C (usually takes at least 10 min).
  b. Aspirate medium of cell culture (cells most easily grown on 1:100 collagen-coated 6-well or 10 cm plates).
    ***Note:*** 60%–90% confluency is recommended for electroporation. A fully confluent well of a 6-well plate holds around 1 million HEK293T cells. A fully confluent 10 cm plate holds around 10 million HEK293T cells.
  c. Add enough pre-warmed 0.05% Trypsin-EDTA into the culture flask to completely cover cells (0.6 mL to a well of a 6-well plate, or 3 mL to a 10 cm plate).
  d. Incubate for 5 min in 37°C cell culture incubator to fully dissociate cells (Figure 1B).
    **CRITICAL:** Efficient transfection requires single cell suspension, which is attained with sufficiently long exposure to Trypsin.
  e. Add equal volumes of DMEM complete, gently mix by pipetting 5–10 times to single cell suspension, then collect the cell suspension into a 15 mL centrifuge tube.
  f. Count the number of cells using hemocytometer (Weber Scientific). Load 12 μL of cell suspension into hemocytometer and visualize using a light microscope. There are four square regions to count cells. Ensure at least 30 cells are in each region for accurate cell counts. After counting all four regions, determine the average number of cells in each square region. Multiply that number by 10,000 to yield the estimated number of cells per mL.
  g. 800,000 cells are required for each reaction. Add the volume of cell suspension that contain 800,000 cells into a separate 15 mL tube, centrifuge at 200 × *g* for 3 min.
  h. Carefully aspirate the supernatant using a pipette, removing as much as possible without disturbing the pellet. Resuspend with 2 mL PBS to wash cells (Figure 1C).
  i. Centrifuge at 200 × *g* for 3 min. Remove as much supernatant as possible using a pipette without disturbing the pellet.

**CRITICAL:** PBS wash is required to completely remove DMEM and trypsin before addition of Cas9/gRNA.

5. Electroporation of HEK293T cells using Lonza nucleofector.
  a. Transfer the 20 μL mixed nucleofection reagent into the cell pellet to form ∼25 μL cell suspension. Mix well by pipetting up and down 5 times.
  b. Make sure that the cell culture hood lights are turned OFF. Then, move RNP mixture into hood and remove aluminum foil covering.
  c. Transfer ∼25 μL cell suspension to the 5 μL RNP mixture; add 1 μL Cas9 EP enhancer (Figure 1D). Mix well by pipetting up and down 10 times.
  d. Transfer all (∼30 μL) to one unused well of the 16-well electroporation cuvette using a P20 pipette.
    **CRITICAL:** Carefully add the liquid to avoid formation of bubbles. One or two bubbles is not a problem.
  e. Go to 4D-Nucleofector and enter settings: select the cuvette well intended for electroporation in the machine touchscreen. Next, select “Cell Line SF” from the list of options. For the electroporation code, use CA-189 for HEK293T cells (Figure 1E). Press “OK” on touchscreen.
  f. This brings up the next screen (Figure 1F). The 4D-Nucleofector opens a slot that allows placement of the cuvette. After placing the cuvette in the holder, confirm the settings and touch “START” on touchscreen to electroporate.
    **CRITICAL:** Ensure that the cuvette is placed in the right orientation in the nucleofector. The yellow pin must be visible on the side closest to the nucleofector body (Figure 1G). If the strip is mounted in the wrong orientation, the yellow pin is hardly visible because the cuvette will not be flush in the holder.
    **CRITICAL:** Use some method of labeling which wells have already be used for electroporation. This can be done by labeling the cuvette cap accordingly, but please ensure that the orientation of the cuvette cap is consistent relative to the cuvette.
    ***Note:*** White precipitate may appear after electroporation and is not cause for concern. If anything, it is a visual cue that the electroporation worked.
  g. Appearance of a green ‘+’ indicates successful electroporation (Figure 1H). After electroporation, go back to culture hood with lights OFF, add 50 μL of PBS into cuvette, transfer all to 250 μL PBS in an Eppendorf tube for ∼330 μL total.
  h. Add 50 μL each to three wells of 48-well plate (A, B, C) (Table 2).

**Table 2:** Organization of cell samples after electroporation

Well A	Well B	Well C
Light right after delivery	Light 1 h after delivery	No light applied
Indels measured at 3 days	Indels measured at 3 days	Indels measured at 3 days
  i. Add 300 μL “DMEM complete” media only to wells B and C (Figure 2A).

**Figure 2:**
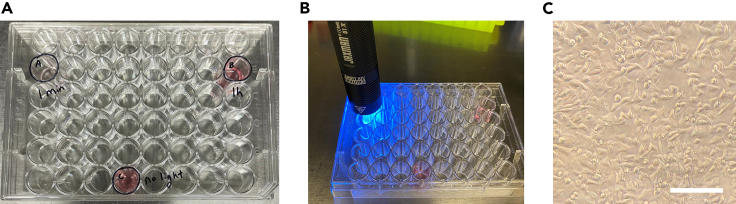
Light illumination for deactivation of Cas9/pcRNA (A) Sample A has 50 μL of PBS. Sample B and C have 50 μL PBS + 300 μL DMEM complete. (B) Sample A is illuminated by the LED flashlight for 1 min. The flashlight is balanced and centered on the well. To avoid unintended illumination of multiple wells, we suggest separating each well into different 48-well plates. For this protocol, multiple wells were used in the same plate for ease of presentation in the figure. (C) Cells in Sample A one hour after electroporation and light illumination. Cells are healthy and already adhering to the well. Scale bar corresponds to 100 μm.

### Deactivation of Cas9/pcRNAs using light illumination

**Timing: 30 min**

6. Deactivation right after electroporation to well A (containing 50 μL cells in PBS).
  a. Turn on 365 nm LED flashlight, hold it 2 cm above well A for 1 min (Figure 2B). Cells in well A are now deactivated.
  b. Add 300 μL “DMEM complete” media to well A. Cover plate with aluminum foil, move back to incubator.

7. Deactivation at 1 h after electroporation to well B.
  a. By 1 h after electroporation, HEK293T cells should be adherent if collagen is coated properly (Figure 2C).
  b. First carefully remove most of the media from well B using a pipette. Be sure to not disturb the recently adhered cells.
  c. Turn on 365 nm LED flashlight, hold it 2 cm above well B for 1 min.
    **CRITICAL:** DMEM complete can partially block the light, which greatly reduces deactivation efficiency.
  d. Add back 300 μL fresh “DMEM complete” media. Cover plate with aluminum foil, move back to incubator.

### Harvest genomic DNA (gDNA) and detect indel mutations via sanger sequencing

**Timing: 3 h**

Genomic DNA from HEK293T cells exposed to various conditions are purified, then subject to PCR to amplify ∼400 bp product that includes the edited region of the genome. Approximately 10 ng of genomic DNA input is sufficient for high quality PCR that accurately quantifies editing efficiency using Sanger Sequencing.

8. Harvest genomic DNA (gDNA) from HEK293T cells.
  a. 3 days after electroporation, wash cells off 48-well plates with 200 μL PBS, using pipette or cell scraper.
    ***Note:*** Between 10,000 to 1 million cells should be sampled to yield enough gDNA and properly estimate the frequency of indels. In this experiment, there are approximately 100,000 cells initially plated to each 48-well after electroporation, which increases to approximately 300,000 after 3 days of cell growth and division.
  b. From cells suspended in 200 μL PBS, purify genomic DNA (gDNA) using DNeasy Blood & Tissue Kit following manufacturer’s protocol. Elute in 100 μL AE supplied with kit. Store gDNA in −20°C.

***Optional:*** Measure DNA concentration using the NanoDrop 2000 UV-Vis spectrophotometer. Estimation of DNA concentration should be greater than 10 ng/μL.

**Pause point:** continue with PCR any time.

9. Perform PCR to amplify region around Cas9 target site. The forward and reverse primers used are *HEKs4_F* and *HEKs4_R*, respectively, from Table 1.
  a. Combine for PCR reaction. Mix by pipetting up and down 5–10 times.

**Table:** 

Component	Volume
Nuclease Free Water (NFW)	3 μL
Genomic DNA (10–50 ng)	1 μL
Fwd/Rev primer set (5 μM)	1 μL
Q5® High-Fidelity 2**×** Master Mix	5 μL
**Total**	**10 μL**
  b. Start thermocycling protocol (for HEKs4_F/HEKs4_R primer set)

**Table:** 

PCR cycling conditions			
Steps	Temperature	Time	Cycles
Initial Denaturation	98°C	30 s	1
Denaturation	98°C	10 s	35 cycles
Annealing	68°C	10 s	
Extension	72°C	20 s	
Final extension	72°C	2 min	1
Hold	4°C	Forever
  c. Extract PCR-amplified target DNA with QIAquick PCR Purification Kit following manufacturer’s protocol. The following reagents except 3 M sodium acetate (NaAc) are supplied with the kit. Briefly, adding 50 μL PB (binding buffer), 10 μL of 3 M NaAc, mix using a pipette, then loading a spin column. Spin in microcentrifuge at 17,900 × *g* for 1 min, decant the collection tube, then load 750 μL PE (wash buffer). Spin with same protocol, decant the collection tube, then spin again to dry spin column membrane. Elute in 30 μL EB.
    ***Optional:*** Measure DNA concentration using the NanoDrop 2000 UV-Vis spectrophotometer. Estimation of DNA concentration should be greater than 1 ng/μL.
    ***Optional:*** Run PCR product on gel electrophoresis. We use E-Gel® EX Gel, 2% pre-cast gels on the E-Gel® iBase™ Power System/E-Gel™ Safe Imager™ Real-Time Transilluminator. Running PCR product on gel electrophoresis should yield a band of the expected size.
  d. Submit 10 μL for Sanger Sequencing, using the Fwd and/or Rev PCR primer as the sequencing primer. We use GeneWiz or a core facility at our institution.
  e. Also sequence wild type HEK293T cells for a “negative control” sample with no genome editing.

**Pause point:** continue with Sanger sequencing analysis any time.

10. Evaluate genome editing efficiencies from Sanger sequencing results using TIDE: Tracking of Indels by DEcomposition (Brinkman et al., 2014).
  a. Obtain Sanger sequencing reads in .ab extension format.
  b. Directly visualize the Sanger sequencing traces using a program such as SnapGene.
  c. Visit the TIDE website.
  d. Enter GGCACTGCGGCTGGAGGTGG as the guide sequence for *HEK site 4.*
  e. Enter the .ab sequencing file for the negative control under “Control Sample Chromatogram (.ab1 or .scf)”
  f. Enter the .ab sequencing file for the intended sample under “Test Sample Chromatogram (.ab1 or .scf)”
  g. Press the blue “Update View” button. The results will automatically update on the main website panel.

## Expected outcomes

**Figure 3:**
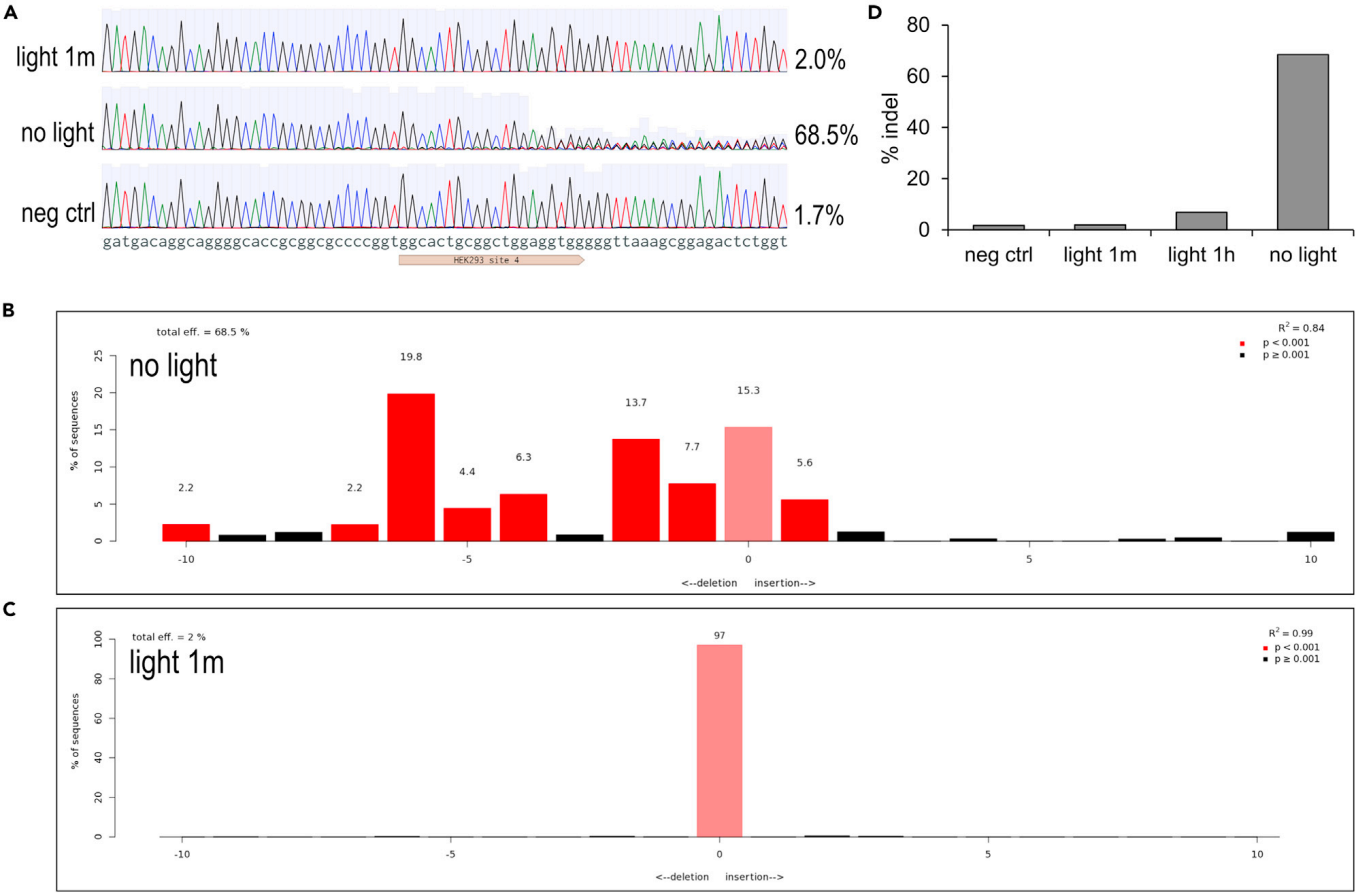
Sanger sequencing and indel measurement (A) Sanger sequencing traces around the Cas9 target site for cells exposed to Cas9 followed by light right after electroporation (‘light 1m’), cells exposed to Cas9 but no light (‘no light), or cells without Cas9 exposure (‘neg ctrl’). 2.0%, 68.5%, 1.7% correspond to the estimated indel percentage at 3 days after electroporation for each sample. (B) Output of TIDE indel analysis for cells exposed to Cas9 but no light (‘no light’). (C) Output of TIDE indel analysis for cells exposed to Cas9 followed by light right after electroporation (‘light 1m’). (D) Quantification of indel percentage at 3 days after electroporation for the samples described in panel a. Quantification for cells exposed to Cas9 followed by light at 1 h after electroporation is also included (‘light 1h’).

Direct visualization of Sanger sequencing traces (.ab1 files) should show a single dominant nucleotide signal at each base pair position for the samples without Cas9 (‘neg ctrl’) and with Cas9/pcRNA but light illumination as soon as possible after electroporation (‘light 1m’). In contrast, for samples with Cas9/pcRNA and not exposed to light, i.e., the Cas9 inside cells remains active (‘no light’), the base pair positions downstream of the target sequence will have variable nucleotide signal, indicating a mixture of different sequence species due to presence of indels (Figure 3A).

Entering the Sanger sequencing traces in TIDE (Brinkman et al., 2014) will estimate the identity of each indel product in the mixture. For the ‘no light’ sample, there is a wide distribution of deletions between 1 to 10 nucleotides, or an insertion of a single nucleotide (Figure 3B). For the ‘light 1m’ sample, there are almost no detected indels, because the cells had its intracellular Cas9/pcRNA deactivated before they had a chance to induce genome editing (Figure 3C). Quantification of expected indel results for all samples are shown (Figure 3D).

## Limitations

This protocol is reliable for all cell types compatible with electroporation using the Lonza system. We have successfully tested this protocol on HEK293T, K562, U2OS, and WTC-11 iPSCs with some variations to the protocol. We have mainly validated Cas9 deactivation after delivery of Cas9 protein in complex with light-deactivatable pcRNA. Because the pcRNA cannot be genetically encoded, delivery using other strategies such as lentivirus is likely infeasible. Direct delivery of pcRNA via lipofection is also feasible, but we found was significantly less efficient compared to electroporation.

The method for indel quantification using Sanger sequencing also has limitations. First, it is unable to detect lower levels of indels below 2%–3% due to the noise of Sanger sequencing. Second, there are some variations in quantification compared to other methods for indel measurement such as high-throughput sequencing and the T7E1 assay (Sentmanat et al., 2018). Sanger sequencing was chosen for its convenience, but other methods such as high-throughput sequencing can detect lower levels of indels (even below 0.1%) without potential miscalling of indels.

## Troubleshooting

### Problem 1

Electroporation protocol on the Lonza machine yields an error, represented by a red ‘–’ in the case of multiple errors or a yellow ‘+’ in the case of few errors (“warning”), rather than the green ‘+’ that indicates electroporation success (step 5g).

### Potential solution

We have never obtained electroporation errors with HEK293T cells but have encountered such errors on occasion using other cell types such as K562. To resolve these errors, select the problematic well on the graphical display to obtain more details including an error breakdown. Refer to the 4D-Nucleofector™ System Manual for a comprehensive explanation of each error code. Empirically, we found that a couple of changes may increase the chance of success: 1) decant as much PBS as possible from the cell pellet before nucleofection buffer is added – this increases the relative volume of nucleofection buffer compared to cells, Cas9, other buffers, etc. 2) Try the electroporation protocol using different numbers of cells. Reducing the number of cells may improve likelihood of success. 3) Ensure that bubbles are not introduced when transferring the cell mixture into the cuvette before electroporation. Excessive bubbles can also cause the electroporation to fail.

### Problem 2

HEK293T cells are not appropriately adherent by 1 h after electroporation or look otherwise unhealthy (step 7a).

### Potential solution

First, ensure that the collagen is fresh. Ensure that the tissue culture plate material is polystyrene and packaged with pre-treatment that makes the surface more amenable to cell attachment.

If the cells look unhealthy, one possibility is unintended temperature shock. Ensure that the trypsin, cell media, or PBS used are completely preheated to 37°C prior to removal from water bath/heating unit, sprayed with 70% ethanol, wiped with paper towels, and moved to inside the hood.

Finally, rule out mycoplasma infection as a cause of unexpected changes in cell morphology by performing mycoplasma testing.

### Problem 3

Sanger sequencing failed for various reasons (mispriming, sequencing noise) (step 10a).

### Potential solution

Run PCR product on gel before Sanger sequencing to verify presence of a single amplicon. If not, then the PCR failed and the PCR protocol should be further optimized. Another check is to ensure that Sanger sequencing works on samples without Cas9 exposure (genomic DNA from unmodified cells).

### Problem 4

No detected indels from Sanger sequencing in samples with Cas9/pcRNA delivered and without light illumination, despite successful electroporation (green ‘+’ observed on the 4D-Nucleofector after electroporation) (step 10g).

### Potential solution

First, verify that there are indels when the pcRNA is replaced with the wild type gRNA version of the target sequence. This involves performing the same electroporation experiment detailed in the protocol, but replacing the pcRNA (*Pcl5_HEKsite4* in Table 1) with the wild type gRNA (*gRNA_HEKsite4* in Table 1) version.

If there are indels when using the wild type gRNA, then the pcRNA itself may be problematic. This could be because all the pcRNA were already deactivated due to accidental exposure to sub 400 nm wavelength light illumination, the ordered target sequence was incorrect, or the company incorrectly synthesized the gRNA. To avoid accidental exposure to sub 400 nm wavelength light, be sure to cover the pcRNA whenever possible with aluminum foil, though exposure to ambient light is unlikely to cleave the pcRNA. Furthermore, during the light illumination protocol, separate each condition into a different plate so that light illumination to one well does not bleed through to adjacent wells, or use black wells that prevent light penetration or reflection.

If there are still no indels when using the wild type gRNA, the problem could arise from improper electroporation, defective Cas9, or defective tracrRNA.

### Problem 5

Light illumination of cells with Cas9/pcRNA does not prevent indel formation (step 10g).

### Potential solution

Ensure that the path of illumination from the flashlight to the cells is not inhibited by any plastic covering (such as plate cover) or excessive cell media (which could partially absorb the light). Finally, ensure that the illuminating flashlight is fully charged. Try increasing the intensity or duration of the light exposure to see if that results in any deactivation.

## Resource availability

### Lead contact

Further information and requests for resources and reagents should be directed to and will be fulfilled by the lead contact, Taekjip Ha (tjha@jhu.edu).

### Materials availability

This study did not generate new unique reagents.

## Data Availability

Original data have been deposited to Mendeley Data: https://doi.org/10.17632/w55kctmzzc.1. The published article includes all remaining data generated during this study.
